# Mercury Ingestion

**DOI:** 10.5811/cpcem.2018.7.39408

**Published:** 2018-09-05

**Authors:** Manish Amin, Alex Huang, Sudha Challa, Phillip Aguìñiga-Navarrete, Laura C. Castro, Donya Sarrafian

**Affiliations:** Kern Medical, Department of Emergency Medicine, Bakersfield, California

## CASE PRESENTATION

**Image 1:**
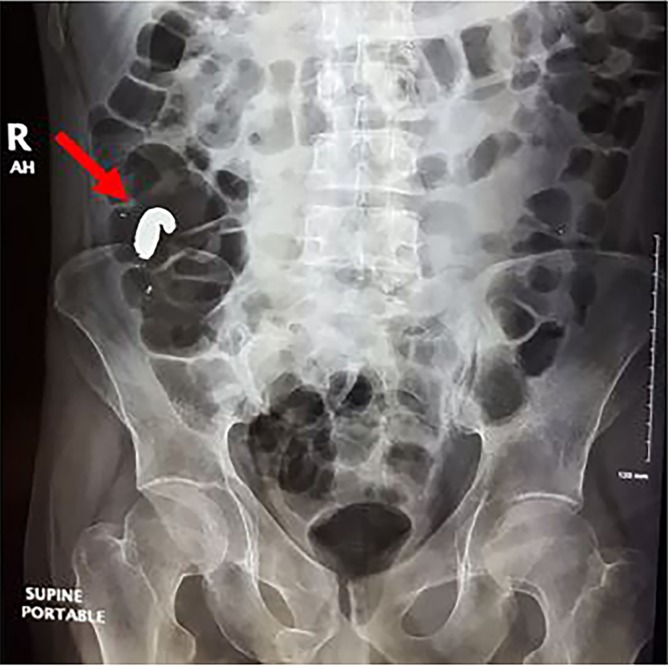
Abdominal radiograph demonstrating high-density material in the right hemi-colonic region (red arrow), which was confirmed to be in the appendix on computed tomography.

**Image 2:**
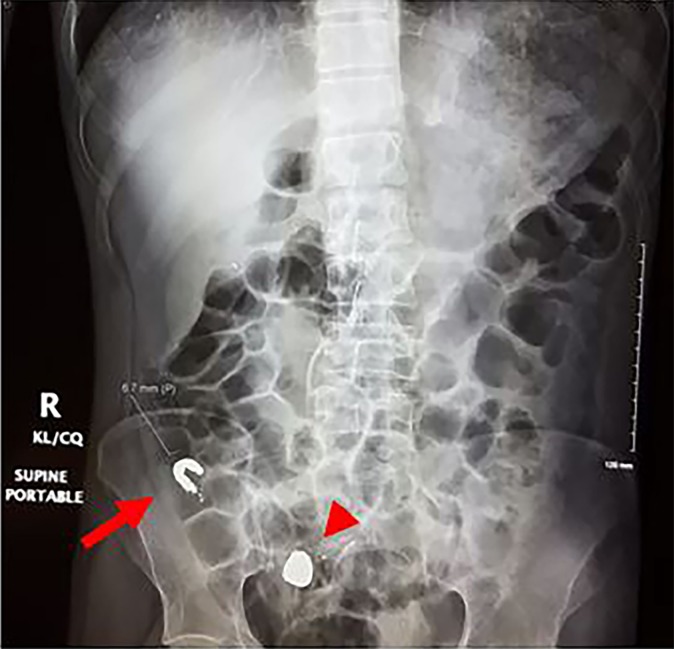
Abdominal radiograph demonstrating an interval new collection of the mercury in the right hemi-pelvis (red arrowhead) and interval decrease in the collection of mercury in the appendiceal lumen (red arrow).

A 30-year-old male with history of bipolar disorder and previous incident of elemental mercury ingestion in a suicide attempt, presented to the emergency department with new-onset nausea and vomiting. Abdominal radiograph showed a collection of metallic material in the appendix (Image 1), which was confirmed by computed tomography. Blood mercury level was found to be 120 micrograms per liter (mcg/L). Repeat abdominal radiograph approximately six hours later, after the patient was placed in lateral decubitus position and Trendelenburg position, showed partial spillage of the mercury out of the appendix into the cecum (Image 2). The patient was admitted for bowel irrigation with chelation therapy. Symptoms resolved after the first day, and repeat radiographs showed gradual clearance of mercury from the colon.

## DISCUSSION

This case demonstrates a successful positioning maneuver of placing the patient in lateral decubitus and Trendelenburg position, which led to significant passage of the retained mercury from the appendix. No other images in the literature demonstrate this characteristic of elemental mercury. Mercury exists in three forms: elemental, inorganic, and organic. Elemental mercury can cause pulmonary toxicity when vapor is inhaled, but it has poor gastrointestinal absorption when it is ingested and is usually excreted over several days with low risk of systemic toxicity.^1^,^2^ However, there have been several case reports of ingested mercury found to be retained in the appendix of patients, which led to the development of appendicitis.^3^ Prophylactic appendectomy vs. conservative management has been described for retained mercury in the appendix.^4^,^5^

**CPC-EM Capsule**

What do we already know about this clinical entity?

Elemental mercury has poor gastrointestinal absorption, but it can become retained in the appendix and lead to appendicitis.

What is the major impact of the image(s)?

It demonstrates that successful patient placement in left lateral decubitus and Trendelenburg position may help dislodge retained mercury from the appendix.

How might this improve emergency medicine practice?

Conservative management with patient positioning and bowel irrigation can be used for patients with mercury retained in the appendix prior to considering surgical intervention.

Documented patient informed consent and/or Institutional Review Board approval has been obtained and filed for publication of this case report.
